# Proposed Integrated Control of Zoonotic *Plasmodium knowlesi* in Southeast Asia Using Themes of One Health

**DOI:** 10.3390/tropicalmed5040175

**Published:** 2020-11-20

**Authors:** Jessica Scott

**Affiliations:** College of Public Health and Medical and Veterinary Sciences, Australian Institute of Tropical Health and Medicine, James Cook University, Townsville 4811, Australia; Jessica.scott2@my.jcu.edu.au

**Keywords:** Zoonotic diseases, Integrated vector management, vector-borne disease, One Health

## Abstract

Zoonotic malaria, *Plasmodium knowlesi*, threatens the global progression of malaria elimination. Southeast Asian regions are fronting increased zoonotic malaria rates despite the control measures currently implemented—conventional measures to control human-malaria neglect *P. knowlesi’s* residual transmission between the natural macaque host and vector. Initiatives to control *P. knowlesi* should adopt themes of the One Health approach, which details that the management of an infectious disease agent should be scrutinized at the human-animal-ecosystem interface. This review describes factors that have conceivably permitted the emergence and increased transmission rates of *P. knowlesi* to humans, from the understanding of genetic exchange events between subpopulations of *P. knowlesi* to the downstream effects of environmental disruption and simian and vector behavioral adaptations. These factors are considered to advise an integrative control strategy that aligns with the One Health approach. It is proposed that surveillance systems address the geographical distribution and transmission clusters of *P. knowlesi* and enforce ecological regulations that limit forest conversion and promote ecosystem regeneration. Furthermore, combining individual protective measures, mosquito-based feeding trapping tools and biocontrol strategies in synergy with current control methods may reduce mosquito population density or transmission capacity.

## 1. Introduction

Malaria is a vector-borne parasitic infection that is endemic in tropical and subtropical climates. At the end of 2018, an estimated 228 million cases occurred internationally (95% CI: 231–278 million), with ongoing indigenous cases reported from 81 endemic countries [1].

The Southeast Asia region has reported a 70% reduction in human-malaria infection incidence rates between 2010 to 2018 [1]. While the region closely approaches elimination, the lack of consideration towards disease-specific management strategies of *Plasmodium knowlesi* hinders the prospect of total human-malaria elimination [2]. Simian-malaria, *P. knowlesi*, is recognised as the fifth species capable of causing human malarial disease [1,2,3]. Before the rise of molecular-based diagnostics, human infections with *knowlesi*-malaria was thought to be improbable [4]. A report from the Kapit division of Malaysian Borneo in 2004 [5] detailed the repeated misidentification of *P. knowelsi* infections in humans. The researchers revealed that over half of historical cases recorded between 2000–2002 were misdiagnosed as *P. malariae* by conventional microscopy [4,6]. Since then, incidence and risk rates of *P. knowlesi* malaria have been increasing, and the disease distribution is growing throughout Southeast Asian regions, particularly on the Malaysian mainland [3,7,8]. Cases of natural human infection by *P. knowlesi* has been reported in communities from Malaysia [5,8], Thailand [9,10,11], Cambodia [12], Myanmar [13], Singapore [14], Indonesia [15,16], Vietnam [17] and the Philippines [18,19]. Indigenous *P. knowlesi* cases in Malaysia have spiked considerably between 2016 and 2018 from 1600 to over 4000 cases reported by the World Health Organization [1]. Despite the decreasing prevalence rate of other human malaria-Plasmodium species in Peninsular Malaysia and East Malaysia, the prevalence of *P. knowlesi* is increasing [7,8,20], given that knowlesi-malaria accounted for 70% of reported cases between 2013–2017 [20]. Infection with *P. knowlesi* can progress from an uncomplicated to a severe and potentially fatal condition. This is held attributable to its short 24-hours erythrocytic replication cycle [21,22]. Approximately 1 in10 patients infected with *P. knowlesi* present with or develop severe symptoms [23]. Severe symptoms are fatal in approximately 0.15–1.2% of cases in high endemic areas [20]. In Malaysia, malaria-related deaths were partially attributable to *P. knowlesi*, with mortality rates spiking in 2017 [20]. Most reports of *P. knowlesi* infection are of cases presenting with clinical symptoms; however, asymptomatic infections also occur [24,25]. 

The geographical distribution of *P. knowelsi* is suspected to be confined to Southeast Asian countries corresponding to the presence of both the natural host, the macaque monkey (long-tailed & pig-tailed) and the mosquito vector, the *Leucosphyrus* group [4,6,26,27]. Predominantly in Southeast Asia, *Macaca fascucularis* (*Mf*) and *Macaca nemetrina* (*Mn*) are confirmed natural hosts of *P. knowlesi*. These species are known to co-exist with a varying ecological distribution that constitutes their travel preference [28]. *Mn* is considered a ground dweller, occupying areas of lower human population density and absence from deforested regions [26]. In contrast, *Mf* is a tree dweller that can occupy a wide range of habitats [29] with sightings ranging from urban settlements, wetlands and crop farms [30]. Several definitive-host mosquito species are incriminated with the ability to carry *P. knowlesi* in their salivary glands. These mosquito species belong to members of the Leucosphyrus group of *Anopheles*. Vectors that have been implicated are *An. balabaecensis*, *An. latens*, *An. cracens*, *An. donaldi*, *An. dirus*, *An. sundaicus* and *An. introlatus* (Table 1). Each species is described to inhabit a vast range of ecotypes, favouring areas with dispersed forest cover, with biting tendencies favouring outdoors as opposed to indoors [26]. 

Current control measures to eliminate malaria are directed towards chemically reducing vector transmission and establishment of infection through the integration of long-lasting insecticide-impregnated bed nets (LLINs) and indoor residual spraying (IRS) of houses [1]. Despite the success of current control strategies, they are insufficient for *P. knowlesi* control, as they neglect the parasite’s ongoing transmission between the host-animal reservoir population. The consequence of an animal reservoir threatens the prospect of human-malaria elimination as there is a risk of continuous residual transmission [4,7]. 

This review will reflect on the genetic subpopulations of *P. knowlesi* across humans and the natural hosts, the distribution and behavioral changes of the vectors and natural hosts in response to the pressure of ecological changes, which are potentially permitting *P. knowlesi* transmission to humans. Investigating the complex dynamics of *knowlesi*-malaria transmission at an ecological interface of the human-animal-environment paradigm allows for designing and optimizing effective disease control strategies that deviate from current conventional approaches. This review will also highlight potential strategies to reduce mosquito contact rates and population densities in human settlements through describing alternative changes to land-use, utilizing personal protective clothing in communities, implementing vector trapping and baiting tools coupled with future direction towards biocontrol alternatives. These strategies are primarily directed towards reducing the contact of human populations to the animal reservoirs and incriminated vector species.

## 2. Ecological Drivers of *Plasmodium knowlesi* Transmission

### 2.1. The Significance of P. knowlesi Subpopulations in Human Disease Transmission

Zoonotic mammalian, *Plasmodium knowlesi*, shares a conserved evolutionary lifecycle, including a definitive *Anopheles*-mosquito host and nonhuman primate and now human intermediate hosts for successful completion of its sexual reproductive cycle. It remains uncertain whether human infection with *P. knowlesi* has resulted from a recent host-switch event from macaques to humans, resulting in human-vector-human transmission or is currently co-existing and circulating among human and macaque populations [41]. It is speculated that both transmission dynamics are occurring simultaneously, but for now, it appears macaque-vector-human is the primary route of infection transmission [42,43]. 

The genetic evolution of *Plasmodium spp*. has occurred over millenniums, often evolving parallel with their respective host species [41]. It is presumed that a successful host-switch event of the Plasmodium parasite across into mammalian hosts occurred over 64MYA [41]. Genetic sequence analysis of *P. knowlesi*, mtDNA data from Sarawak, Malaysia indicate that ancestors of *P. knowlesi* persisted in macaques before the time of *Homo Saipans* colonization, also exemplifying that the parasite population underwent significant expansion approximately 30,000–40,000 YAGO [21]. There is currently limited evidence supporting that host-switch events from macaques to humans have played a central role in the diversification of *P. knowlesi* in Southeast Asia [41,44]. 

Genetic diversity analyses of *P. knowlesi* isolates in Peninsular Malaysia and East Malaysia has uncovered population substructures that infect humans and macaque reservoirs [45,46,47]. Presently, three divergent subpopulations of *P. knowlesi* are classified in East and West Malaysia. The first subpopulation described is predominantly associated with long-tailed (*Mf*) macaques, the second is related to pig-tailed (*Mn*) macaques. Both subpopulations are widespread in East Malaysia; it is hypothesized that these subpopulation clusters are sympatric, genetically isolated by geographical distance, and maintained due to the high differentiation across the genome [45,46,48]. In contrast, the third cluster is more commonly associated with humans than macaques, primarily detected in Peninsular Malaysia [45,47]. The significant differentiation of genetic structure between the two macaque-associated clusters and the Peninsular Malaysia cluster indicates allopatric divergence. This is potentially due to the ocean barrier between these locations, which has restricted the migration of the macaque hosts [47]. The *P. knowlesi* cluster associated with *Mf* dominates human infection cases in East Malaysia, exhibiting an intense selection pressure, while the cluster related to *Mn* is common in Sarawak [43,46]. 

Whole-genome sequencing of *P. knowlesi* isolates revealed recent recombination events occurring between Peninsular Malaysia clusters and Malaysian macaque *Mf*-*P. knowelsi* and *Mn*-*P. knowlesi* clusters [48,49]. Benavente and colleagues [49], have identified that *P. knowelsi* subpopulation clusters in Peninsular Malaysia have acquired fragments of erythrocytic invasion genes, including DBPβ and NBPXa protein encoded regions from the *Mn*-*P. knowlesi* associated cluster. In line with these findings, genetic exchange of the NBPXb gene was detected in 27% (*n* = 33) of *Mn*-*P. knowlesi* isolates that were identically present in *Mf-P. knowlesi* subpopulations [49]. Moreover, researchers also found significant SNP variation across five genes related to reticulocyte binding and Duffy binding protein genes involved in erythrocyte invasion from the Peninsular Malaysia cluster when compared to the other two macaque associated clusters [49]. It is not confirmed whether the genetic exchange events between these clusters affect transmission dynamics between the macaque reservoirs and human populations. Still, it is speculated that this exchange could improve binding and invasion efficiency in the erythrocytic phase of *P. knowlesi*’s lifecycle [49]. In a separate study, recent chromosomal segment exchanges were detected between Malaysian Borneo *Mf*-*P. knowlesi* subgroups and *Mn*-*P. knowlesi* subpopulations [50]. These exchanges potentially relate to genes associated with *P. knowlesi’s* interaction with mosquitoes during its lifecycle [50].

Specifically, on chromosome 8, Benavente et al. [50], described that the *Mf* associated *P. knowlesi* genotype from Borneo Malaysia had two distinct geographical sub-groups that correspond with Kapit and Betong regions. Exemplifying significant chromosomal variation between geographical locations which has led to the further subdivision of cluster one [50]. The researchers demonstrated that chromosome 8 was identical between the *Mn-P. knowlesi* cluster and the Betong subgroup of *Mf-P. knowlesi* associated cluster, which also appears to be under strong selective pressure [50]. This finding presumes that *P. knowlesi* is adapting to the two macaque hosts ecology with enough genetic differentiation to be considered separate subgroups, capable of recombining if parasite populations overlap [48,50]. Determining whether diversification patterns result from adaptations to a new host and/or environment remains a complex issue to address [44]. In Sabah, Malaysia, a casual association between increased incidence of *knowlesi*-malaria infections and progressive environmental modifications was hypothesized, suggesting that malaria is sensitive to environmental changes such as deforestation [51,52]. 

### 2.2. The Influence of Deforestation, Changing Land-use and Risk of P. knowlesi Transmission

Exposure to human *P. knowlesi* infection occurs in regions where the vector and natural host species are proximately present [26,27]. Local ecological disruptions such as deforestation and biodiversity loss are potential drivers for the increased associations between humans and the reservoir host and vector, influencing the transmission dynamic of *knowlesi*-malaria infection [29,51,52,53,54,55,56]. Understanding the influence and mechanisms of biodiversity loss in ecosystems and increasing the risk of infectious disease acquisition is a multi-factorial issue [56,57,58]. A systematic review highlighted that on a regional scale, there was a positive correlation between enhanced richness of human infectious diseases and a high-level of animal biodiversity and population density in the Asia-Pacific and a negative correlation between vector-borne disease outbreaks and forest cover. Conversely, the research gathered from the same study also concluded that zoonotic-specific outbreaks were positively correlated with the number of threatened animal species and forest cover. While decreased forest cover does not increase vector-borne disease outbreaks, the findings imply that a loss of forest cover and biodiversity increases the risk of zoonotic disease outbreaks [58]. It is hypothesized that the effects of deforestation or reduced forest cover are reducing animal populations and biodiversity, which is likely resulting in a loss of the ’dilution effect’ or the ’wasted transmission’ notion between non-specific hosts and the zoonotic pathogen spread [57,58,59]. The nature of biodiversity loss and zoonotic pathogen transmission risk is variable depending on the disease system, local ecology, and human social behavior. Inferring that disease risk is likely a local scale phenomenon that relies on reservoir hosts and vector-specific compositions and ecology [57]. 

At a local scale in Malaysian Borneo, ~80% of the forest landscape has undergone deforestation or agriculture expansion between 1990 and 2009, leaving only 3–8% of protected forests intact [60]. Deforestation in this region is typically attributable to the rapid development of industrial plantations [61], with this trend likely to continue [62]. When considering these factors, it highlights the potential consequences of decimating ecosystems and the increased threat of zoonotic disease outbreaks [58]. It also reflects the social and economic realities that allow for the rapid conversion of forests to industrial land [63]. The modification of local forested landscapes for anthropogenic use has been shown to produce favorable environments for prominent mosquito populations harboring *P. knowelsi* by generating forest fragments and fringes [31,54,55]. Disruption of the local forests due to deforestation also substantially impacts macaque host-reservoirs population distribution and density [29,52], resulting in their encroachment into human settlements [40]. The progressive loss of habitat diversity and increased forest fragmentation, and decimation due to deforestation has conceivably influenced the prevalence of *P. knowlesi* and altered the behavior and transmission dynamics between the macaque, the vectors and humans [58,59,64]. 

### 2.3. The Influence of Forest Disturbances on the Mosquito Vector and Transmission Dynamics

*Plasmodium knowlesi* is propagated and transmitted to humans by several definitive host-mosquito species, involving broad members from the Leucosphyrus Group of *Anopheles* (Cellia) (Diptera, Culicidae) [26], summarised in Table 1. Broadly, the members of the Leucosphyrus group, including the *Dirus* and *Leucophyrus* complexes, are predicted to occupy areas with high coverage of disturbed forests that typically lack intact forest cover [26,55]. Members of the *Leucosphyrus* complexes span vast biogeographical locations. Variations in mosquito distribution, biting behavior and ecological adaptions are also speculated to result from progressive deforestation [55], as Anopheline mosquito populations richness and diversity have been reported to decrease from the forest edge to plantations through to human settlements [55]. An extensive analysis reviewing vector data across twelve countries and 87 mosquito species found that 56.5% of confirmed mosquito vectors of human infectious diseases were positively associated with deforestation [54]. It was demonstrated in this study that vector behavior, distribution and abundance are potentially favored in deforested locations. The downstream effects of deforestation have been described to alter mosquito breeding sites, abundance, species composition, differences in resources, predation, nutritional reserves, and survival. All of which may support the vector’s ability to transmit zoonotic *P. knowlesi* in human populations and circulate among macaque populations. 

Several studies have demonstrated vector composition changes concerning geography and season to explain transmission variations [30,32,55]. Collectively, Leucosphyrus mosquitoes complex known to transmit *P. knowlesi* display commonalities in their biting behavior and host preferences for both humans and macaques. *An. balabacensis* is considered a significant vector of knowlesi-malaria in parts of East Malaysia, spanning across various habitats. The abundance of infected *An. balabacensis* has been reported more frequently at forest edges than village settlements [32,55], even though the biting rate of humans was higher in housing settlements, outdoors around the household [30,65]. In addition, this vector has also been found in logged forests, palm oil plantations, farms and playgrounds [31,32]. *An. Balabacensis* has a biting behavior that displays a greater predisposition to transmit *P. knowlesi* to humans in secondary forest locations and farmland rather than human settlements [30,65]. The high frequency of human feeding coupled with life expectancy estimates, has potentially contributed to the high vectorial capacity to transmit this parasite to humans. Like *An. latens*, this vector species has been captured during the early evening to early morning in village settlements, caught significantly outdoors, around the perimeter of houses rather than inside [30,32,34,66]. *An. latens* in Sarawak, Malaysia, had human biting rates highest at the forest fringe, followed by the forest and at human settlements [33,34]. It was also reported that its tendency to transmit the parasite to humans was highest in farms [34]. In Selangor, Malaysia, *An. introlatus* has been incriminated to transmit *P. knowlesi*, but no sporozoites were detected in the salivary glands, only oocytes in the mosquito’s midgut. Its propensity to transmit the infection to humans was considered low due to a low biting rate [40]. Indicating that it is unlikely that this species is a competent vector of *P. knowlesi* [41]. A significant vector of *P. knowlesi* transmission in Peninsular Malaysia from the Dirus complex is *An. cracens*. *An. cracens* has a clear biting preference for humans rather than macaques outside human settlements, with peak biting between 7 pm and 9 pm [67]. Its tendency to bite monkeys at ground level or in the canopies where the macaque reservoir resides indicates it is a significant reservoir to facilitate *P. knowlesi* transmission [35]. The ability of *An. cracens* to transmit this parasite to a new susceptible host were approximately two-fold greater in fruit orchard locations than forests due to its high biting rate in humans [35]. 

### 2.4. The Influence of Forest Disturbances on the Natural Host, Prevalence and Transmission Dynamics

In Southeast Asia, the natural hosts of *P. knowlesi*, *Macaca fascicularis* the long-tailed and *Macaca nemestrina*, the pig-tailed macaques, occupy various biogeographical regions and ecotypes [26,27,68]. *Macaca fascicularis* (*Mf*) is principally a tree traveler, inhabiting forests with intact forest cover, while *Macaca nemestrina* (*Mn*) prefers to travel along the ground [26,28]. Traditionally, these two species’ geographical ranges are described to overlap but are often found separated into wetter alluvial rivers and dry hilly terrains, respectively [28]. Macaque behavioral adaptations through habitat modification have increased their sightings in urban areas and forest fringes near human settlements. Specifically, *Mf* has been detailed to occupy a variety of habitats ranging from urban areas, forest fragmentations, croplands and wetlands, as well as forested areas. Contrariwise, *Mn* has been predicted to favor forested areas away from human settlements [26]. 

Prevalence rates of *P. knowlesi* infection within the *Mf* and *Mn* populations demonstrate heterogeneous spatial distribution respective to their geographical locations [68,69]. High prevalence of Macaque parasite infection has been reported in Selangor Malaysia, Sarawak, Malaysian Borneo with lower prevalence described in Pahang, Perak, Johor and Kuala Lipis states in Peninsular Malaysia as well as in Singapore, Philippines, Provinces in Thailand and Laos (Table 2). In Sarawak, Malaysia, from 2004–2008, 108 *Mf* and *Mn*, caught 2km from large human settlements, had positive cases of *P. knowlesi* with an infection ratio of 87% (*n* = 83) and 50% (*n* = 26), respectively [21]. Wild macaques caught in forested regions tend to have greater portions of *P. knowlesi* infection relative to Macaques that were caught peri-domestically [70] and from wildlife national parks [71]. These findings highlight that macaque species dwelling in forested locations are more susceptible to increased contact with incriminated mosquito species, as there is overlap in mosquito resting and breeding sites [69]. Recently, Funguang and colleagues [72] have reported a natural infection of *P. knowlesi* in *Macaca arotdes*. *Macaca arotdes* populations are found in forested areas in the continental Southeast Asian countries, including but not limited to Myanmar, Thailand, Vietnam, Laos and Peninsular Malaysia [72], suggesting that there may potentially be an overlap with known vectors and other natural hosts [26,68].

Local ecological changes attributable to deforestation and land-use change devastate forested habitats and disrupt ecological regulation and function [64]. Such devastation within macaque populations has been speculated to cause a change in home-ranging behavior and distribution, which has consequently caused them to occupy new habitats and associate closely with humans [26,29]. Stark and colleagues [29] followed a *Mf* macaque troop for several months to observe behavioral adaptations to deforestation events. During active deforestation periods, the troop size dissipated, and home range locations became dispersed. Leaving the macaque troop to occupy new habitats before equilibrating [29]. The findings represented here demonstrate that local-scale environmental changes to macaque habitat structure may impact infectious disease transmission dynamics due to the disruption in macaque home-range behavior that may cause them to inhabit human settlements [29,73]. Describing the true nature of *P. knowlesi* prevalence in Southeast Asia is perplexing to estimate, as there is no consistent information in patterns of infection and exposure in any given location. In Southeast Asia, maps of human environmental susceptibility to *P. knowlesi* have been generated based on known data relating to anthropogenic land-use, the geographical distribution of reservoir species, vector and reported *P. knowlesi* infections in humans, macaques and mosquitoes [26,27,68]. Risk assessment index mapping has the potential to be used to focus investigations in undefined locations that would aid in completing the picture of host and vector compositions and their respective distribution to ascertain accurate transmission dynamics between the humans and natural hosts.

### 2.5. The Influence of Individual Behavioural Factors that Increase Risk of Disease Acquisition

Host risk factors highlighted in recent epidemiological studies have found that adult males are more likely to acquire *P. knowlesi* infection than females [8,20]. Assessment of human and environmental factors associated with *knowlesi*-malaria risk found that interactions and sighting of macaque species, predominantly *Mf*, was associated with an increased risk of acquiring *P. knowlesi* infection [66]. The findings also support a consistent risk factor profile for *knowlesi*-malaria with men working as subsistence farmers, especially those who work in palm-oil plantations, with the highest individual transmission risk occurring at the forest fringe [19,61,66,77]. Investigations into peri-domestic transmission and exposure to *P. knowelsi* in Sabah, Malaysia, found that the primary vector, *An. balabacensis* was caught significantly around household areas rather than inside household areas, by five-fold [65]. In line with these findings, Grigg and colleagues [66] uncovered that while spraying household walls with residual insecticides reduced the likelihood of *P. knowelsi* infection**, the utility of LLINs did not appear to provide sufficient protection against disease acquisition [66]. In this study, it was also found that the acquisition of *knowlesi*-malaria was strongly associated with community members that traveled during the night and slept outdoors [66], leading to speculation surrounding the efficacy of LLINs and IRS applications for *P. knowelsi* transmission control. These findings indicate that individual-level risk profiles related to behavior and interactions with the environment have a considerable impact on the acquisition risk of *knowlesi*-malaria infection. It also highlights the limitations of using LLINs for *P. knowlesi* transmission control. 

## 3. Integrative Control Perspectives

The challenges that *knowlesi*-malaria presents to malaria elimination efforts are the increased presence and proximity of reservoir hosts to humans and the outdoor blood biting nature of the vectors that transmit the infection [30,34]. Deforestation and forest fragmentation have unequivocally been attributable to the proximal movement of the macaque reservoirs and vectors to humans [27,29]. Human encroachment onto historically forested areas has increased the risk of exposure to outdoor transmission of *P. knowlesi*. Especially for outdoor, occupational workers (e.g., plantation and agricultural workers) [77,78,79]. The transmission dynamics of *P. knowlesi* at the moment appears to be primarily macaque-vector-human. Although, human-vector-human transmission is also likely occurring in communities as well, due to the movement of outdoor workers to urban areas [20,42,43,66]. Thus, a transdisciplinary approach through targeting vectors contact with humans must be actioned, deviating from current conventional malaria control methods.

### 3.1. Improved Disease Surveillance of P. knowlesi Infection

Public health sectors in Southeast Asia should begin focusing on managing the underlying transmission processes of *P. knowlesi* based on a comprehensive empirical understanding of disease ecology and epidemiology to appropriately implement control measures. Establishing a strategic plan to control *knowlesi*-malaria must first correctly estimate disease occurrence [26]. A quantitative approach to surveillance should address the influence of local-scale spatial variation to the landscape and reservoir population transmission hot-spots and dynamics to provide insight into the mechanistic links between human incidence and land-use change to support the integration of interventions [80]. Additionally, to support and focus on implementing control interventions, further investigations into the bionomics and blood indices of incriminated mosquito vectors are also warranted to determine the distribution and relative risk of *knowlesi*-malaria infection in human populations. Furthermore, monitoring the genetic subpopulations of *P. knowlesi* could provide an understanding of transmission dynamics occurring in urban communities and potentially ascertain whether there is an association between specific genetic subpopulations of *knowelsi*-malaria and clinical outcomes [43,77]. 

### 3.2. Ecological Interventions: Deforestation Regulations and Forest Restoration Strategies

It is presumed that biodiversity acts as a buffer of zoonotic pathogen spread, reducing outbreaks of vector-transmitted disease through a “dilution” type effect or perhaps a “wasted” transmission notion to non-susceptible hosts [58,59]. Nevertheless, local and international economic trade pressures, specifically from palm oil plantations, are just one of the significant contributors to rapid deforestation and degradation of biodiversity in Southeast Asia, with demands for this commodity set to increase [62]. Local government environmentalists could action alternatives to address the threats imposed by agricultural businesses potentially through: regulating against the conversion of forests to palm oil farms, penalizing those promoting non-certified palm-oil products and promoting alternative, biodiversity-friendly uses of forested land [63]. Furthermore, methods to increase biodiversity and ecosystem function are required, focusing on mammal conservation. In particular, forest restoration is necessary for these forest fragments and in underproductive oil-palm plantations to increase available habitat for wildlife with a further endorsement to allow either the regeneration or assisted enrichment of plants needed to support the movement of threatened wildlife in these areas [64]. 

### 3.3. Individual-level protection: Insecticide Treated Clothing

Current control interventions of malaria in Southeast Asia rely on the adequate coverage of LLINs and IRS to decrease malaria transmission risk [1]. These methods have been proven successful for human-malaria transmission control, although it is insufficient to control the residual transmission of zoonotic *P. knowelsi* [66]. Vectors capable of transmitting *knowlesi*-malaria have biting behavior that is exophagic, with peak biting times during the early hours of the morning and evening [30,34]. Consequently, this mosquito behavior increases the level of exposure to knowlesi-malaria for community members with outdoor occupational duties and those partaking in night-time forest activities [19,78]. Therefore, there is a need to implement access to individual-level protective measures for high-risk occupational groups. This approach requires a partnership between the government health sectors, plantation and agricultural industries and local communities [81]. 

Repellents such as topical repellents, Insecticide Treated Clothing (ITC) and spatial repellents are protective measures currently available against outdoor-biting mosquitoes. However, ITC is not easily accessible in endemic malaria communities [82]. In a systematic analysis of various repellents, it was found that although topical and spatial repellents can provide some level of personal protection, there is insufficient evidence to propose that these repellents can reduce malaria incidence [83,84]. Topical repellents are not widely accepted, primarily because adequate protection requires high user compliance with re-application needed every couple of hours [83,85]. Furthermore, its wide-scale efficacy in different settings has not been addressed for this measure to be deployed as a viable intervention strategy [86]. Standardized application protocols of the repellents for optimal efficacy have not been ascertained yet [84].

Alternatively, ITC offers a more robust and acceptable intervention for malaria control that has the potential for large-scale distribution in highly endemic areas, which could be incorporated in occupation clothing for industrial plantations and agricultural land workers [81,82]. ITC are routinely deployed to military personal staying in high endemic regions of arthropod-borne infectious diseases [87,88,89]. It is reported as effective against bites from *Anopheles* and *Adese* mosquitoes [87,90,91]. Reporting on the efficacy of ITC is heterogeneous and should be interpreted with caution, as there is currently no standardized testing or reporting methods presently being used to compare efficacy [87]. The effectiveness of ITC appears to either demonstrate no protective capacity at all [92] or can potentially reduce the risk of malaria infection by 50% [84] to 70% [93]. Despite ITC potential for reducing zoonotic and human malaria, further research is warranted, particularly regarding its scalability, cost feasibility, length of durability, safety under variable environmental working conditions, resistance to washing and UV light exposure [82,89,94].

### 3.4. Vector Behavioural Tools: Synthetic Odour-based Traps and Baits for Control of Vector Transmission of P. knowlesi

LLINs and IRS are integral control measures to mitigate the transmission risk of human Plasmodium infection inside households. However, this does not account for mosquitoes that bite outside living quarters during the early mornings and evenings when community members do not use their bed nets. Odor-based traps such as the human-landing catches (HLC) are currently available to lure mosquitoes based on their feeding behaviors. Recently Van De Straat et al. [95], evaluated a synthetic odor-based trap that is fan-powered, baited with CO2 plus an appropriate odor lure that mimics a human host. The trap enables indoor and outdoor utility, reducing the risk of exposure to potentially infectious mosquito bites. Relative to the HLC, this trap is safer and cheaper. However, its complexity and operational constraints, like the requirement for CO2, limit its scalability to be accessible for general area-wide vector control strategies [96]. A novel based approach incorporated the technique of odor attractants without the requirement of a trap. Instead, this odor attractant is a bait laced with a semiochemical, termed the ’attract-and-kill’ strategy [96]. The attractive bait is sugar-based, which attracts both mosquito sexes, making it in-discriminant of blood-host preferences, increasing its utility of outdoor usage. The lacing of the toxicant increases the mosquito’s mortality rate, reducing health risks and impacts on non-target species while potentially decreasing resistance development [96]. 

### 3.5. Biocontrol Strategies Targeting Vector Transmission

Supplementary to environmental and localized action, biocontrol strategies offer an environmentally friendlier and cheaper alternative that targets different stages of the mosquito life cycle by exploiting their behavior. Biocontrol strategies may increase mortality within mosquito populations or render the mosquito incapable of transmitting the infection [97]. However, the introduction of symbiotic bacteria as potential agents for mosquito transmission control is practically challenging. Still, recent success integrating the bacteria *Wolbachia* into the *Adese agypti* mosquito populations in North Queensland, Australia, has reduced this stigma. *Wolbachia* may be a potential lead for malaria control, but speculation concerns whether *Wolbachia* should be utilized for infection in *Anopheles* mosquito populations. A recent review has emphasized the potential risk of enhancing *Plasmodium* infection in mosquitoes [98]. Nonetheless, this risk is restricted to specific Plasmodium and host-reservoir species with no research yet on the risk of *Wolbachia* increasing *P. knowlesi* infection displayed by *Anopheles* mosquitoes [98].

Entomopathogenic fungi is a potentially promising vector biopesticide [99] that is isolated from similar breeding habits to mosquitoes [100]. The fungal species can infect the mosquito through contact resulting in penetration into the mosquito’s midgut. The fungal agent acts by disrupting the mosquito’s microflora and obscures the parasite’s progression from oocysts to sporozoites [101] and accelerates mosquito mortality [102,103]. Mnyone et al. [104], described potential delivery applications by utilizing various surfaces such as walls and polyester netting, testing for efficacy and persistence to malaria-infected mosquitoes. The researchers found that entomopathogenic fungal species increases daily mortality rates and represents a control measure that could be utilized alongside LLINs and IRS [104] as well as the lacing capacity of the sugar-baiting notion described previously. The scarcity of studies that describe fungi’s effects on mosquito populations indicates that further research is needed to determine the viability, infectivity targets, and persistence of fungal spores in the mosquito population [104]. Particularly concerning its interactions in Southeast Asia Anopheles mosquito populations and *P. knowlesi.*

Recently, Wang et al. [105] have bioengineered a bacterium *Serratia AS1* with recombined strains that allow it to secrete anti-plasmodium effector proteins, reducing parasitic load by 93%. Thus, reducing the capacity of the mosquito to transmit the infection [106]. In laboratory conditions, the bacterium could spread through mosquito populations via vertical, horizontal and transstadial transmission [106], exhibiting potential for long-term persistence in wild mosquito populations, having no ill effect on the mosquito’s lifecycle or environment [106]. Acquisition of mosquito infection by the modified bacteria has been shown achievable through mechanisms like larval diet and larval breeding waters [107], exemplifying the potential of incorporation into components of the ’attract-and-kill’ strategy. However, questions about *Serratia AS1* potential for off-target infection, viability under deployed field conditions, the feasibility of large-scale commercial production, the potential for evolution of resistance and reproducibility in Anopheles mosquitoes infected with *P. knowlesi* has yet to be investigated.

## 4. Conclusion and Integrative Control Proposal

In Southeast Asia, *P. knowlesi* malaria challenges the prospect of malaria elimination. Conventional control measures of malaria transmission have proven effective to an extent. Nevertheless, given the zoonotic nature of *knowlesi*-malaria, these measures neglect an ongoing transmission capacity between the mosquito-vector and natural-macaque hosts to humans. The events that have conceivably contributed to the increased incidence of *P. knowlesi* infection in humans are potentially a result of habitat modification and anthropogenic changes to land use. This factor has likely contributed to genetic exchange events between *P. knowlesi* subpopulations and increased human exposure to potentially infective macaque-reservoirs and mosquito-vectors. Implementing control strategies that mitigate *knowlesi*-malaria transmission to humans should be scrutinized at the human-animal-environment interface, deviating from current conventional measures. Surveillance efforts that accurately address disease transmission and distribution among humans, macaques, and mosquito populations are required to inform these interventions. Individual-level personal protective clothing is likely to be a measure that is feasible and acceptable. However, standardized, community-based studies are needed to determine its efficacy against malaria transmission. Biocontrol strategies offer an eco-friendly, safe, and sustainable alternative to reduce the density of mosquito populations, granted that there is still a scarcity of research that addresses the viability and feasibility of the potential organisms under field conditions. Exploiting mosquito feeding tendencies using mosquito behavioral-based feeding tools and traps could work alongside proposed and current measures but its overall efficacy in reducing human P. *knowlesi* incidence in communities has yet to be thoroughly investigated.

## Figures and Tables

**Table 1:** The Occurrence of Described Vectors of *Plasmodium knowlesi*.

Vector	Geographical Location	Common Areas of Capture	Biting Behaviour	Host Preference	Author
*An. balabaecensis*	Sabah and Sarawak, Malaysia, Kuala Lipis, Philippines	Village sites, forest edges, small farming sites, logged forest areas, palm oil estates, shrub bushes	Exophagic & endophagic, acrodendrophillic	Humans and Macaques	[30,31,32]
*An. latens*	Sarawak, Malaysia	Forests, forest fringe, farms and longhouses	Acrodendrophillic, Exophagic	Humans and Macaques	[33,34]
*An. cracens*	Pahang and Sarawak, Malaysia, Thailand, Indonesia	Village sites, fruit orchards	Exophagic, ground-level biting	Humans and Macaques	[35,36]
*An. dirus*	Sarawak, Malaysia, Southern Vietnam	Forest and Forest fringes	Exophagic and acrodendrophillic	Not described	[37]
*An. donaldi*	Sarawak, Malaysia	Village sites, farm huts, long-houses	Not described	Not described	[38]
*An. sundaicus*	Katchal island, India	Village sites	Not described	Not described	[39]
*An. introlatus*	Selangor, Malaysia	Farmlands, forests	Exophagic & endophagic.	No comparison made	[40]

**Table 2:** Prevalence and Geographical Occurrence of *Plasmodium knowlesi* Infection in Macaque Host Species.

Country	Findings	Reference
Peninsular Malaysia, Pahang & Kuala Lipis	145 *M. fascicularis* blood samples were collected. In Pahang, *P. knowlesi* was detected in 6.9% of samples. In Kuala Lipis, 77 *M. fascicularis* blood samples were collected. *P. knowlesi* was detected in 7.3% of samples.	[67]
Peninsular Malaysia, Pahang, Perak and Johor	103 *M. fascicularis* blood samples were collected. In Pahang, *P. knowlesi* prevalence among *M. fascicularis* was 26.5%. In Perak, *P. knowlesi* prevalence among *M. fascicularis* was 3.8%. In Johor, *P. knowlesi* prevalence among *M. fascicularis* was 2.6%.	[71]
Kapit Division of Malaysian Borneo	108 macaque blood samples were collected. In 83 *M. fascicularis*, 82 were positive for plasmodia infection. *P. knowlesi* prevalence was 87%. In 26 *M. nemestrina*, only 21 were positive for plasmodia infection. *P. knowlesi* prevalence was 50%.	[21]
Selangor, Malaysia	70 *M. fascicularis* blood samples were collected*. P. knowlesi* prevalence among *M. fascicularis* was 60%. Four out of 35 had mono-infection of *P. knowlesi*. Co-infection with multiple simian Plasmodium species occurred in 65% of samples.	[74]
Thailand	In 93 blood samples collected from three macaque reservoir hosts, *P. knowlesi* prevalence was 2.5%. It was detected only in *M. arctodes.*	[72]
Loas	276 *M. fascicularis* samples were collected from various regional populations across Southeast Asia. One P*. knowlesi* infection was detected in Laos. The prevalence of *P. knowlesi* in across the Southeast region was 0.4%.	[75]
Thailand	Retrospective analysis of blood films from *M. fascicularis* from 2006-2009. Prevalence of *P. knowlesi* has increased within *M. fascicularis* across the Northern-western, Eastern and South provinces of Thailand.	[10]
Gulf of Thailand	In 195 *Macaca fascicularis*, 5.7% were infected with *P. knowlesi*, and in 449 *Macaca nemestrina*, 2.3% were infected with P. knowlesi.	[76]
The Philippines, Palawan island	95 *M. fascicularis* blood samples were collected. *P. knowlesi* was detected in 19% of cases.	[69]
Singapore	65 peri-domestic and 92 wild macaques blood samples were collected. It was found that the former group was uninfected, while 71.7% of the sampled wild macaques were positive for at least one simian-malaria parasite species. *P. knowlesi* had a prevalence of 68.2% among the infected wild macaques.	[70]
